# The relationship between parents’ sleep quality and sleep hygiene and preschool children’ sleep habits

**DOI:** 10.5935/1984-0063.20220051

**Published:** 2022

**Authors:** Azita Chehri, Paraveh Taheri, Habibolah Khazaie, Amir Jalali, Alireza Ahmadi, Reza Mohammadi

**Affiliations:** 1 Department of Psychology, Kermanshah Branch, Islamic Azad University - Kermanshah - Iran; 2 Sleep Disorders research Center, Kermanshah University of Medical Sciences - Kermanshah - Iran; 3 Substance Abuse Prevention Research Center, Institute for Health, Kermanshah University of Medical Sciences - Kermanshah - Iran; 4 Department of Caring Science and Society, Div of family Medicine and Primary care, Karolinska Institute - Karolinska - Sweden; 5 Department of Anesthesiology, Critical Care and Pain Management, Imam Reza Hospital - Kermanshah University of Medical Sciences - Kermanshah - Iran

**Keywords:** Sleep Hygiene, Child Day Care Centers, Parents, Sleep

## Abstract

**Objective:**

The relationship between parents’ sleep quality and sleep hygiene and preschool children’s sleep habits was examined.

**Material and Methods:**

The study population consisted of the parents of all preschool children at daycare centers located in Kermanshah Province. Through cluster sampling, 153 parents from 26 daycare centers were selected. In order to assess the quality of sleep and sleep health of parents, Pittsburg sleep quality index (PSQI) and sleep hygiene index (SHI) were used. As to the status of childrens sleep habits (CSH), the parents also completed child sleep habit questionnaire (CSHQ). Data analysis was performed in SPSS version 25. The non-parametric tests like U Mann-Whitney test, Kruskal-Wallis’s test, Spearman’s rho, and regression test were used.

**Results:**

The mean score of CSHQ from the parents’ viewpoint was 56.34±7.96, which meant a relatively improper sleep habits in the children. The CSH was significantly and directly related to parents’ sleep quality and all of its subscales except two sub-scales (SSQ and HSE). In addition, CSH was directly and significantly related to the parents’ sleep hygiene and its three subscales. The results of multiple linear regression showed that the impact coeffcient of parents’ sleep quality (B=1.02), given the t-value, predicted changes in CSH with 0.99 confidence.

**Conclusion:**

In general, the results showed that CSH was in a relatively bad state, while it had a direct relationship with parents’ sleep quality and hygiene. To improve CSH, it is possible to improve parents’ sleep quality through implementing proper programs and motivating parents to observe sleep hygiene.

## INTRODUCTION

Sleep is one of the essential and vital needs of human, with a notable effect on regaining power, growth, energy saving for body metabolism, brain function, neural maturity, learning skills, and memory^1^. In fact, sleep plays a key role in children’s development^2^ so that it affects physical, behavioral, and emotional growth and also cognitive performance of children^3^. Childhood is a critical stage in everyone’s life in which the foundations of wellbeing and a life-long health are formed. Sleep health during childhood is a key element, which is mostly neglected^4^. The prevalence of short sleep duration, sleep behavioral problems, and sleep-disordered breathing is 20-50% in children between three to five years old^5^. The symptoms and side-effects of inadequate sleep are different between children and adults and they are expressed as mood and activity level change, daytime sleepiness, irritability, behavioral problems, learning difficulties, motor vehicle crashes in teenagers, and poor academic performance^6^. Li et al. (2013)^7^ reported that children and adolescents’ sleep time has decreased by 0.75min/night in year over the past decades. It is estimated that 15%-75% of children at school age do not have adequate sleep.

In fact, creating a healthy sleep pattern in children depends on informing parents and their cooperation. Therefore, children’s sleep quality must be part of every health check and the parents must know that creating a healthy sleep pattern is as important as good nutrition, and teeth hygiene^8^. In the same way, sleep habits and behaviors reflect cultural differences^3^ and parents’ life condition and behaviors affect child’s condition. Any problem in this regard might lead to formation of permanent unhealthy habits in one’s life^9^. Studies have shown that with a decrease in parents’ sleep time or a delay in their sleep, unhealthy sleep habits in children increases^10^. Therefore, sleep hygiene and healthy pattern in parents affect sleep condition in children^11^. Ronnlund et al. (2016)^10^ concluded that parents with sleep problems had children with significantly higher sleep problems. Children with adequate sleep have better performance and they are at a lower risk of mood and behavioral disorders^12^. Therefore, diagnosing and preventing improper sleep habits during childhood are highly important^13^. Clearly, without efficient strategies to improve sleep quality, sleep patterns usually diverge from healthy sleep norms^3^. Taking into account that one of the priorities of sleep hygiene is to determine quality of sleep and measure the level of observing sleep hygiene in society and that these factors have a key role in formation of sleep habits in the future generations, the present study is an attempt to determine the relationship between sleep habits of preschool children and sleep quality and sleep health of their parents in Kermanshah. Based on the above, two hypotheses were designed:

Hypothesis one: there is a direct and significant relationship between daycare children’s sleep habits in Kermanshah City and their parents’ sleep quality.

Hypothesis two: there is a direct and significant relationship between parents’ sleep hygiene and children’s sleep habits.

## MATERIAL AND METHODS

The present study was carried out as an analytical and cross-sectional work in 2019 on 153 preschool children selected through cluster sampling. Kermanshah city was divided into 10 districts and five clusters were randomly selected and from each cluster five daycares were randomly selected and out of each, 10 children were selected through convenient sampling. Out of a total of 250 children, the parents of 170 children were willing to participate in the study and only 153 series of questionnaires were used. By definition, preschool children in Iran are in the age range of 4.5 to 6 years who have not entered their seventh years of life by the 22^nd^ September of the year.

Among the inclusion criteria were preschool age range, parents’ consent and cooperation with the study team, no history of surgery, no congenital, metabolic, and mental disorder disease, not using medicine, and residing at Kermanshah City. In addition, data gathering was done using demographic form and standard questionnaires as follows:

### Pittsburg sleep quality index (PSQI)

The questionnaire was introduced in 1989 by Buysse et al.^14^ to measure sleep quality and help determining sleep quality of individuals. The scale is a self-statement tool with seven subscales. In PSQL scoring, seven components should be considered. The minimum and maximum scores for each component are zero (no problem) and three (very serious problem), respectively. The total score is the sum of scores of each component that ranges from 0 to 21. A high score of each component or total score indicates a poor sleep quality. The sum of the scores of the seven scales constitutes the total score, which is from zero to 21. An overall score of 6 or higher indicates a poor sleep quality^14^. The questionnaire has been validated in Iran with a Cronbach’s alpha equal to 0.83 and correlation coefficient of 0.88^15^.

### Sleep hygiene index (SHI)

The questionnaire is a 13-item self-statement index to measure environmental and behavioral variables that might lead to an unhealthy prolonged sleep. The index was designed by Mastin et al. (2006)^16^ and each item is a five-alternative scale (always, often, sometimes, rarely, and never). Each item is rated on a five-point scale ranging from zero (never) to four (always). The total score ranges from zero to 52, with a higher score representing poorer sleep hygiene^16^. The psychometrics specifications of the tool with three subscales have been examined in Iran with Cronbach’s alpha equal to 0.83, 0.81, and 0.79. The minimum and maximum scores of the tool are 0 and 52, respectively, and the higher the score the lower the sleep hygiene^17^.

### Child sleep habit questionnaire (CSHQ)

The questionnaire was introduced by Owens et al. (2020)^18^ with 45 items and eight subscales to measure sleep quality and habits in children. Here, only 33 items were relevant and filled out by the parents. The questionnaire is designed for children in 4-12 age range and the items are designed in eight subscales. This questionnaire consists of 45 questions, some of which have only diagnostic and therapeutic value, so that only 33 questions are included for scoring. Each item has a value between 3 and 1 (rarely to usually) with the exception of items (26, 11, 10, 3, 2.1) which are reversely scored. The score range is between 33 and 39. The score of each subscale is the sum of the mentioned questions. The subscales of the questionnaire are sleep resistance (1, 3, 4, 5, 6, and 8) with minimum and maximum scores equal to 6 and 18, respectively. Sleep onset delay (2) with minimum and maximum score of 1 and 3, respectively; sleep duration (9, 10, and 11) with minimum and maximum scores of 3 and 9, respectively. Sleep anxiety (5, 7, 8, and 21) with minimum and maximum scores of 4 and 12, respectively. Night awakenings (16, 24, and 25) with minimum and maximum scores of 3 and 9, respectively. Parasomnia (12, 13, 14, 15, 17, 22, and 23) with minimum and maximum scores of 7 and 21. Sleep disordered breathing (18, 19, and 20) with minimum and maximum scores of 3 and 9, respectively; and daytime sleepiness (26, 27, 28, 29, 30, 31, 32, and 33) with minimum and maximum of 8 and 24, respectively. The higher the score of sleep habits questionnaire, the more intense are the sleep problems (18)^18^. The questionnaire has been validated in Iran with Cronbach’s alpha equal to 0.8^19^.

### Data analysis

Data analysis was performed in SPSS version 25. According to the results of Kolmogorov-Smirnov test, the data of interval variables did not follow a normal distribution and non-parametric tests like U Mann-Whitney test, Kruskal-Wallis’s test, Spearman’s rho, and regression test were used. According to the studied interval variables, multiple linear regression was used to determine the prediction of children’s sleep habits based on parental health and sleep.

## RESULTS

Among the 153 participants, 74 were girls (48.4%) and 79 were boys (51.6%). In addition, the surveys showed that the mean ages of the mothers and fathers were 34.8±5.66 (23-53) and 39.71±6.53 (27-61) years, respectively. The mean age of the children was 5.43±0.37 (5-6) respectively. In terms of number children in each family, 29.4% of the families only had one child, 58.8% had two children, and 11.8% had three or more children (each family had only one child at the age under consideration, and the other children in families with two or more children were older than seven years or younger than three years). With regard to financial status, 19% had a good financial status (1,500$<), 75.2% had an average financial status (1,500$-1,000$) and 5.9% had a poor financial status (<$500) (Table 1).

**Table 1:** Demographic characters of research participants.

Variables	N (%)
**Gender (children)**	
Girl	74(48.4)
Boy	79(51.6)
**Educational statue (fathers)**	
Elementary	14(9.2)
High school diploma	53(34.6)
Higher education	86(56.2)
**Educational statue (mothers)**	
Elementary	18(11.8)
High school diploma	49(32)
Higher education	86(56.2)
**Number of children**	
Ono	45(29.4)
Two	90(58.8)
Three & more	18(11.8)
**Child rank in the family**	
First	83(54.2)
Second	60(39.2)
Third and more	10(6.5)
**Family Income (monthly)**	
Less than 500$	29(19)
500-1000$	115(75.2)
More than 1500$	9(5.9)

As listed in Table 2, the mean score of parents’ sleep hygiene is equal to 12.83±6.67 and the mean score of sleep quality in the parents based on Pittsburg scale is 7.76±2.5. In addition, the mean score of sleep habits in the children from the parents’ viewpoint is 56.34±7.96.

**Table 2:** Descriptive of results of CSHQ, PSQI, and SHI in this study.

Index		Min	Max	Mean	SD
Child sleep habit	Sleep resistance	8	17	11.2	2.17
	Sleep onset delay	1	3	2.34	.75
	Sleep duration	3	9	5.84	1.51
	Sleep anxiety	4	12	7.12	2.32
	Night wakings	3	8	4.2	1.21
	Parasomnia	7	18	9.1	1.79
	Sleep disordered breathing	3	8	3.8	1.22
	Daytime sleepiness	8	20	12.76	2.43
	**Child sleep habit (CHS)**	37	82	56.34	7.96
Pittsburg sleep quality (parents)	Subjective sleep quality (SSQ)	0	4	1.95	.52
	Sleep latency (SL)	1	2	1.14	.35
	Sleep duration (SDu)	0	3	.98	.83
	Habitual sleep efficiency (HSE)	0	3	.88	.81
	Sleep disturbances (SD)	0	3	1.27	.5
	Use of sleeping medication (USM)	0	3	.47	.82
	Daytime dysfunction (DD)	0	3	1.06	.77
	**Total sleep quality (TSQ)**	3	17	7.76	2.5
Sleep hygiene (parents)	Sleep and waking cycle behaviors	0	15	5.93	3.41
	Bedroom agents	0	12	1.55	1.98
	Effective behaviors on sleep	0	17	5.35	3.1
	**Sleep hygiene**	1	44	12.83	6.67

Notes: SSQ = Subjective sleep quality; SL = Sleep latency, SDu = Sleep duration; HSE = Habitual sleep efficiency; SD = Sleep disturbances; USM = Use of sleeping medication; DD = Daytime dysfunction; TSQ = Total sleep quality.

As listed in Table 3, the Spearman’s rho showed that the children’s sleep habits had a direct and significant relationship with parents’ quality of sleep in its all subscales except for subjective sleep quality (SSQ) and habitual sleep efficiency (HSE). The parents’ sleep quality also has an indirect and insignificant relationship with sleep onset delay and sleep duration subscales while it has a direct and significant relationship with the rest of children’s sleep habits subscales (sleep resistance, sleep anxiety, night waking, parasomnia, sleep disordered breathing and daytime sleepiness).

**Table 3:** The relationship between child sleep habit and parents' sleep quality in this study.

Variables		Pittsburg sleep quality (parents)							
		SSQ	SL	SDu	HSE	SD	USM	DD	TSQ
**Child sleep habit**	Sleep resistance	.133	.201^*^	.218^**^	.23^**^	.146	.043	.327^**^	.373^**^
	Sleep onset delay	-.84	-.068	-.064	.044	-.116	-.165^*^	-.102	-.149
	Sleep duration	-.031	.007	.053	.05	-.036	.023	-.107	-.001
	Sleep anxiety	-.003	.167^*^	.24^**^	.061	.073	.124	.193^*^	.253^**^
	Night wakings	.163^*^	.094	.102	.19^*^	.28^**^	.244^**^	0.124	.295^**^
	Parasomnia	.049	.125	.27^**^	.12	.264^**^	.33^**^	.226^**^	.383^**^
	Sleep disordered Breathing	.124	.067	.21^**^	.03	.143	.356^**^	.203^*^	.317^**^
	Daytime sleepiness	.038	-.057	.355^**^	.076	.051	.253^**^	-.069	.22^**^
	**Child sleep habit**	.044	.185^*^	.346^**^	.157	.195^*^	.298^**^	.177^*^	.402^**^

Notes: SSQ = Subjective sleep quality; SL = Sleep latency, SDu = Sleep duration; HSE = Habitual sleep efficiency; SD = Sleep disturbances; USM = Use of sleeping medication; DD = Daytime dysfunction; TSQ = Total sleep quality.

* *p*_value_<0.05;

** *p*_value_<0.01.

The results of Spearman’s rho in Table 4 indicates that sleep habits of the children have a direct and significant relationship with the parents’ sleep hygiene and its three subscales. In addition, parents’ sleep hygiene does not have a significant relationship with sleep onset delay and sleep duration, while it has a direct and significant relationship with rest of subscales (sleep resistance, sleep anxiety, night waking, parasomnia, sleep disordered breathing and daytime sleepiness).

**Table 4:** The relationship between child sleep habit and parents' sleep hygiene in this study.

Variables		Sleep hygiene (parents)			
		Sleep and waking cycle	Bedroom agents	Effective behaviors on sleep	Sleep hygiene
**Child Sleep Habit**	Sleep resistance	.126	.076	.191^*^	.173^*^
	Sleep onset delay	-.003	-.211^**^	.163^*^	-.139
	Sleep duration	.067	-.078	.077	.053
	Sleep anxiety	.22^**^	-.093	.143	.22^**^
	Night wakings	.137	.151	.168^*^	.199^*^
	Parasomnia	.263^**^	.255^**^	.116	.28^**^
	Sleep disordered breathing	.237^**^	.202^*^	.238^**^	.28^**^
	Daytime sleepiness	.268^**^	.221^**^	.09	.23^**^
	**Child sleep habit**	.315^**^	.182^*^	.209^**^	.31^**^

* *p*_value_<0.05;

** *p*_value_<0.01.

As listed in Table 5, children’s sleep habits only have a significant and indirect relationship with age, while there is no significant relationship with other demographical variables.

**Table 5:** The relationship between child sleep habit and demographic characteristics.

Variables			Quality of sleep (children)	
		**Mean (SD)**	**Z/R**	**p_value_**
Gender (children)	Boy	57.28 (8.74)	.21	-1.26^*^
	Girl	55.34 (6.98)		
Educational Statue (fathers)	Elementary	56 (8.23)	1.09^**^	.58
	High school diploma	57.32 (8.7)		
	Higher education	55.78 (7.46)		
Educational Statue (mothers)	Elementary	55.1 (5.41)	4.34^**^	.112
	High school diploma	54.9 (8.5)		
	Higher education	57.4 (7.99)		
The child of several families	First	57.51 (8.8)	4.33^**^	.115
	Second	54.39 (6.51)		
	Third and more	58.1 (7.96)		
Family income (monthly)	Less than 500$	56.33 (10.36)	4.56^**^	0.102
	500-1,000$	56.84 (7.74)		
	More than 1,500$	54.25 (8)		
Number of children			-.084^***^	.3
Age of children			-.217^***^	.007
Age of mothers			-.007^***^	.93
Age of fathers			-.11^***^	.17

* U Mann-Whitney test;

** Kruskal-Wallis test;

*** Spearman's rho.

Table 6 represents multiple linear regression and clearly the impact coefficient of parents’ sleep quality (unstandardized coefficient, B=1.02), given the t-value, can predict variations in children’s sleep habits with 99% confidence. The impact has a positive effect, which means that if one unit is added to parents’ sleep quality score, 1.02 unit will be added to their children’s sleep habits. On the other hand, the impact coefficient of parents’ sleep hygiene (unstandardized coefficient, B=0.159), given the t-value, cannot predict children’s sleep habits with 95% confidence.

**Table 6:** Predictive coefficients of predictor variables of sleep-in children.

		Standardized coefficients	Unstandardized coefficients		
**Sig.**	**T**	**β**	**SE**	**B**	**Model**
.0001	23.38		1.98	46.34	Constant
.0001	3.85	.322	.266	1.02	Pittsburg sleep quality (parents)
.112	1.599	.134	.099	.159	Sleep hygiene (parents)

## DISCUSSION

Totally, 153 preschools children’s parents took part in the study. Based on the hypothesis one, there was a direct and significant relationship between daycare children’s sleep habits in Kermanshah City and their parents’ sleep quality. That is, degraded sleep quality in parents led to sleep habits problems in the children. The regression results showed that the parents’ sleep quality coefficient had a positive effect and predicted changes in children’s sleep habits. Ronnlund et al. (2016)^10^ found in their study that parents’ sleep problem had a significant effect on their children’s sleep habits. De Stasio et al. (2020)^20^ found that parents’ psychological and social problems that somehow affected their sleep quality could be related to sleep habits problem in their children. Several studies have reported the effect of children’s sleep problems on their parents’ sleep^10^,^13^,^21^, which is consistent with our findings under hypothesis one. To explain the findings, family system is the main part of children’s lives, and their sleep problems can notably affect their family’s performance and parents’ sleep and day performance in particular. Similarly, parents’ sleep problems and behaviors can affect their children’s sleep. Behavioral treatments that improve sleep in children may also improve the parents’ sleep.

As to the hypothesis two, the results showed a direct and significant relationship between parents’ sleep hygiene and children’s sleep habits. However, regression test results indicated while parents’ sleep hygiene coefficient had a positive impact on children’s sleep habits, it was not a significant predictor of changes in children’s sleep habits. In this regard, Yuwen et al. (2016)^22^ concluded that inadequate sleep and low sleep quality were common in parents and children, while with efficient intervention about parents’ sleep hygiene, it was possible to improve parents and children’s sleep quality. Pyper et al. (2017)^21^ reported that parents’ support of children’s sleep and good sleep hygiene behaviors in parents and family had a notable impact on children’s sleep habits. In other words, parents’ support and behavior about sleep hygiene is a determining and predictor factor in children’s sleep habits. To explain the difference between the present study and other studies, the small sample group is notable as many parents refused to fill-out the questionnaire and there were strict rules in the daycares that limited our chance to find more participants.

The results showed that children’s sleep habits were not significantly related to the parents’ demographical variables and children’s gender. Only children’s age had an indirect and significant relationship. To explain the findings, children’s sleep habits have a strong relationship with the parents’ behaviors and their emphasize on children sleep hygiene^21^. The findings indicated that the parents with sleep problems also had more problems with their children’s sleep. Assessment of children’s sleep by the parents and small sample size can affect the results. Sviggum et al. (2018)^23^ showed that parents had several problems with their children’s sleep behaviors such as going to bed, going into sleep, bedroom light, and so on. They argued that parents need to observe the standards of sleep hygiene by themselves and through this change sleep behavior of their children. Studies have also highlighted parents’ problems as to their children’s sleep behaviors and habits^11^,^21^,^24^. As shown by the results, children’s sleep habits were somehow a function of their parents’ sleep behaviors and hygiene^10^. The majority of studies in this field have highlighted a mutual relationship in this regard. That is, many parents with sleep problems cause sleep problems for their children. Parents’ sleep issues and unhealthy habits can create sleep problems in their children^21^,^23^.

Sleep habits are of the main factors in children’s growth, while childhood has a profound role in the development of healthy sleep habits and other factors^1^. Based on the results, children sleep examination should not be limited to the child and should also include the whole family including the parents. In addition, the results emphasized on the importance of future works on children’s sleep in which parents’ sleep quality and hygiene should be considered as key factors.

### Study limitations

Finding participants for the study was not easy as many parents were reluctant to fill out the questionnaires and there were strict rules in the daycares. Over a 10 months period, only 170 questionnaires were filled-out, out of which 17 questionnaires were incomplete. Therefore, the study was conducted only with 153 questionnaires. In this study, we tried to prevent the selection bias and the samples were selected through convenient sampling method. However, many parents did not want to participate and complete the questionnaires. Answering the questionnaires and participating in the study by the parents was voluntarily and the participants only received a gift along with the questionnaires. In addition, the evaluation of children’s sleep in this study was done based on parents’ views and opinions, and this is one of the important limitations in this study so that the children’s sleep status could have been reported with bias. In addition, according to non-parametric tests, the effect size was not calculated. Similar studies can be performed in other regions and also adolescents can be included.

## CONCLUSION

Although children’s sleep habits were not significantly associated with the two subscales of parental sleep quality, the results showed that sleep habits in children had a significant relationship with the parents’ sleep quality and sleep hygiene. The results showed that CSH was in a relatively bad state, while it had a direct relationship with parents’ sleep quality and hygiene. To improve CSH, it is possible to improve parents’ sleep quality through implementing proper programs and motivating parents to observe sleep hygiene.

## DECLARATION

### Ethical approval

In this research, the ethical considerations including the principles of confidentiality of information, obtaining written informed consent for participating in study, publication and having the right to withdraw from the research at any time were observed. Ethical committee of Kermanshah University of Medical Sciences approved this study (IR.KUMS.REC.1400-738).

### Consent to publication

The datasets used and analyzed during the current study are available from the corresponding author upon reasonable request.

## ABBREVIATIONS

SSQ: Subjective Sleep Quality
SL: Sleep Latency
SDu: Sleep Duration
HSE: Habitual Sleep Efficiency
SD: Sleep Disturbances
USM: Use of Sleeping Medication
DD: Daytime Dysfunction
TSQ: Total Sleep Quality
CSH: Child Sleep Habit
PSQI: Pittsburg Sleep Quality Index
SHI: Sleep Hygiene Index
CSHQ: Child Sleep Habit Questionnaire
